# Emerging Roles of m7G-Cap Hypermethylation and Nuclear Cap-Binding Proteins in Bypassing Suppression of eIF4E-Dependent Translation

**DOI:** 10.3390/v17030372

**Published:** 2025-03-05

**Authors:** Kathleen Boris-Lawrie, Jessica Liebau, Abdullgadir Hayir, Xiao Heng

**Affiliations:** 1 Department of Veterinary and Biomedical Sciences, Institute for Molecular Virology, University of Minnesota, Saint Paul, MN 55108, USA; lieba005@umn.edu (J.L.); hayir003@umn.edu (A.H.); 2 Department of Biochemistry, University of Missouri, Columbia, MO 65211, USA

**Keywords:** cap exchange, CBP80/NCBP3, DHX9/RNA helicase A-responsive structure, epigenetic modification, m2,2,7-guanosine cap, trimethylguanosine cap (TMG-cap)

## Abstract

Translation regulation is essential to the survival of hosts. Most translation initiation falls under the control of the mTOR pathway, which regulates protein production from mono-methyl-guanosine (m7G) cap mRNAs. However, mTOR does not regulate all translation; hosts and viruses alike employ alternative pathways, protein factors, and internal ribosome entry sites to bypass mTOR. Trimethylguanosine (TMG)-caps arise from hypermethylation of pre-existing m7G-caps by the enzyme TGS1 and are modifications known for snoRNA, snRNA, and telomerase RNA. New findings originating from HIV-1 research reveal that TMG-caps are present on mRNA and license translation via an mTOR-independent pathway. Research has identified TMG-capping of selenoprotein mRNAs, junD, TGS1, DHX9, and retroviral transcripts. TMG-mediated translation may be a missing piece for understanding protein synthesis in cells with little mTOR activity, including HIV-infected resting T cells and nonproliferating cancer cells. Viruses display a nuanced interface with mTOR and have developed strategies that take advantage of the delicate interplay between these translation pathways. This review covers the current knowledge of the TMG-translation pathway. We discuss the intimate relationship between metabolism and translation and explore how this is exploited by HIV-1 in the context of CD4+ T cells. We postulate that co-opting both translation pathways provides a winning strategy for HIV-1 to dictate the sequential synthesis of its proteins and balance viral production with host cell survival.

## 1. Introduction

**Figure 1:**
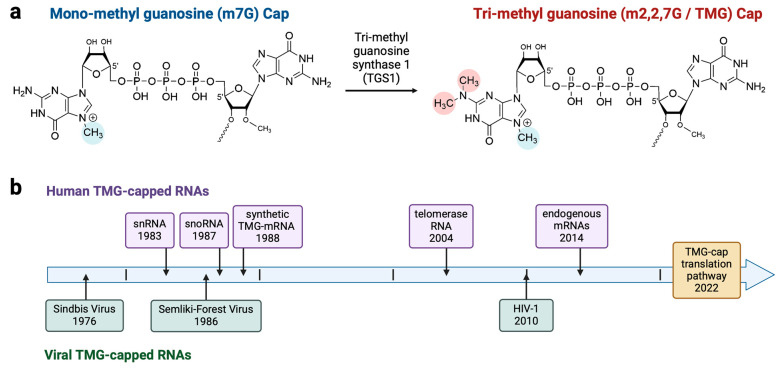
Discovery timeline of TMG-capped RNAs in humans and viruses. (**a**) Chemical structures of mono-methyl guanosine (m7G)- and trimethylguanosine (m2,2,7G, TMG)-caps attached to the transcription start site. Pre-existing m7G-caps are hypermethylated to m2,2,7G-cap by trimethylguanosine synthase 1 (TGS1). Methyl groups: blue, at the 7-position of the m7G-cap; red, at the 2,2 positions of the TMG-cap. (**b**) Timeline of publications. Human RNAs: snRNA in 1983 [10,11,12] and snoRNA in 1987 [13,14]; in vitro translation of synthetic TMG-mRNA in 1988 [15]; telomerase RNA in 2004 [16]; host mRNAs in 2014 [17]. Viral RNAs: Sindbis in 1976 [8]; Semliki-Forest virus in 1986 [9]; HIV-1 in 2010 [7]. Specialized translation licensed by TMG-cap was characterized in 2022 [5].

Historically, HIV-1 (HIV) and other retroviruses have been invaluable models to expose specialized biological processes [1,2,3,4]. Recently, the study of HIV and host mRNAs has identified nuclear cap-binding proteins that drive a specialized translation of mRNAs. This research led to the discovery of a novel pathway, licensed by hypermethylated guanosine cap, m2,2,7G-cap (TMG-cap) [5]. In all domains of life, TMG-cap has critical importance in the assembly of catalytic ribonucleoproteins (RNPs), e.g., ribosomes, spliceosomes, and telomerase [6]. TMG-cap arises from pre-existing mono-methyl guanosine (m7G)-caps, which are acted upon by the enzyme trimethylguanosine synthase (TGS1) (Figure 1a). In 2010, TMG-capping of HIV mRNA was discovered by Yedavalli and Jeang [7]. Studies showed that the HIV transcripts that contain a TMG-cap are the Rev/Rev responsive element (RRE)-dependent mRNAs [7]. In HIV, the TMG-cap is critical for effective viral replication. TGS1 downregulation by siRNA diminishes virion production to 10% of control. Even more significant, the progeny virions are poorly infectious on CD4+ T cells. Compared to control virions of the same quantity, their infectivity is reduced by a factor of 1000 [5]. Therefore, TGS1 activity significantly affects HIV production and progeny virion infectivity.

The discovery of TMG-cap in HIV mRNA added to the previous discoveries of TMG-cap in alphaviruses, which are responsible for vector-borne diseases (Figure 1b); the virological significance of the TMG-cap remains to be determined [8,9].

A persisting barrier to the complete understanding of HIV post-transcriptional regulation has been the role of the viral complex 5′ untranslated region (UTR) in controlling the biogenesis of the complicated viral proteome [18]. The 5′UTR contains many structural domains that coordinate critical events throughout the viral replication cycle. Thermodynamic equilibrium exists between two mutually exclusive conformations of the 5′UTR, which migrate as a monomer or dimer on native gel electrophoresis [19]. Chemical probing, enzymatic probing, and NMR studies have identified distinct base pairings in the monomeric and dimeric 5′UTR conformations [20]. Recent studies have shown that transcription start site heterogeneity significantly affects the 5′UTR conformation and thus the monomer:dimer equilibrium, positing an additional variable in the complicated life of the multi-functional HIV transcript [20,21].

In solution, ribosomal scanning of HIV mRNA structures poses a significant energetic barrier, so the selection of RNAs to translate remains a longstanding issue. The role of ribosome scanning in HIV was made clear by the insertion of upstream AUG codons (uAUGs) at different locations within the HIV sequences. The uAUGs were severely inhibitory to translation in cells and supportive of ribosome scanning rather than internal ribosome entry or ribosomal shunting [22]. Additionally, there is ample evidence of non-canonical ribosome recruitment.

This article reviews the major host translation pathways adapted by HIV. We discuss the evidence that dual control of translation initiation, virus-induced stress, and the mTOR pathway are consequential throughout viral replication. We conclude that HIV-specialized translation likely influences the survival of HIV-infected CD4+ cells.

## 2. Overview of TMG-Cap Biogenesis in Human Hosts

### 2.1. CBC Is a Central Hub for the Maturation of All Pol II Transcripts

RNA polymerase II (Pol II) synthesizes protein-coding transcripts and an abundance of host noncoding RNAs, in addition to retroviral transcripts. The RNAs are processed in a co-transcriptional manner. First, all transcription start sites are modified by the addition of a mono-methylated guanosine (m7G)-cap [23,24]. This guanosine is appended in an atypical 5′ to 5′ linkage and bound by heterodimeric CBP20/CBP80, forming the nuclear cap-binding complex (CBC). The CBC RNA-protein complex tethers the nascent transcript to proteins that carry out splicing and 3′-end processing during the transcription cycle [25,26]. The mature transcript is prepared for CBC replacement by another m7G-cap-binding protein, e.g., TGS1 or eIF4E [27].

### 2.2. Effects of TMG-Cap on Small Nuclear (snRNA), Small Nucleolar RNAs (snoRNAs), and Telomerase RNA (hTR)

The CBC of uridine-rich small noncoding RNAs exchangees to TGS1, as does human telomerase RNA, hTR. TMG-cap facilitates the nuclear retention of snoRNA, which assembles in nucleolar aggregates and assist in ribosome assembly, e.g., U3 snoRNA [28,29]. CRM1 inactivation by leptomycin B (LMB) was shown to trap TGS1 in a complex with m7G-snoRNA [30]. The authors identified that recombinant TGS1 interacted with CRM1 in a Ran-GTP-dependent manner. They postulated that the direct binding of CRM1 to TGS1 promotes TMG-U3 snoRNAs trafficking to the nucleolus [30].

CBC-bound m7G-snRNA recruits ARS2 and PHAX for nuclear export to the cytoplasm via CRM1/RanGTP, where it interacts with SmB and the survival motor neuron (SMN) complex to replace CBC with TGS1. The resulting hypermethylated cap of snRNA is bound by Snurportin, the nuclear import receptor that facilitates the re-import of TMG-snRNA into the nucleus and trafficking to the nucleolus [31,32,33,34].

The noncoding telomerase component-1 RNA (hTR) also acquires a TMG-cap via TGS1 [16,35]. Capping occurs in the nucleus, followed by CRM1-mediated export to the cytoplasm [36]. While not essential for telomerase activity, the TMG-cap of hTR is important for the recruitment of telomerase to telomeres and for the engagement of nuclear Cajal bodies [35]. Failure ofTMG-capping leads to retention of CBC, increased binding of these transcripts to Sm and TERT, and activation of the alternative pathway of telomere maintenance. Also increased telomere elongation is an outcome of diminishing TMG-capping [36]. These emerging studies indicate that TMG-cap is crucial for the regulation of telomere maintenance and telomerase activity.

## 3. Overview of Major mRNA Translation Pathways

### 3.1. In Metabolically Active Cells, the m7G-eIF4E Pathway Is the Predominant Translation Initiation Mechanism

**Figure 2:**
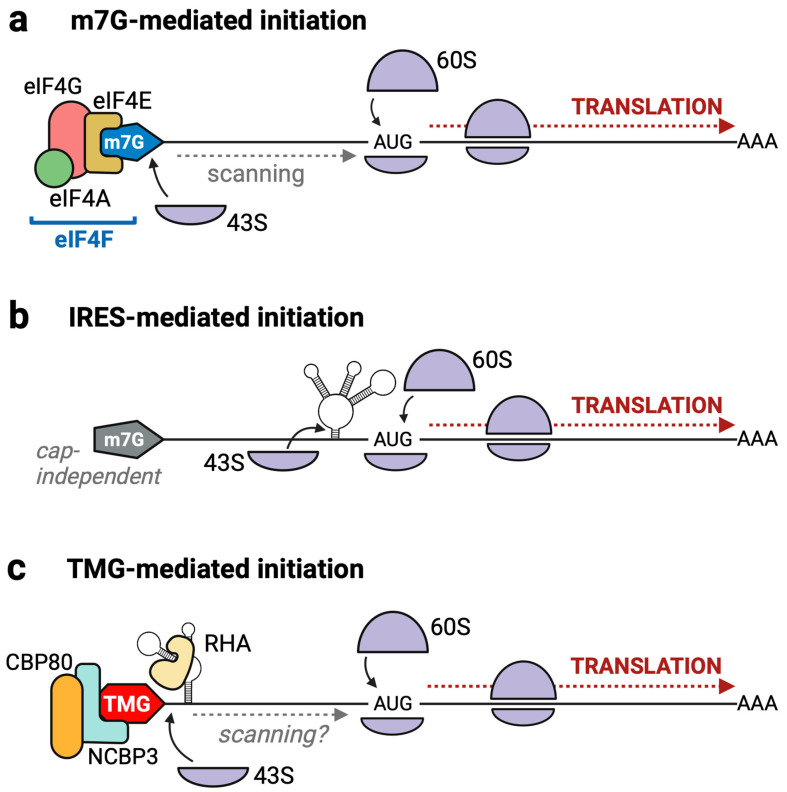
Pathways for host translation initiation. (**a**) eIF4E binds the m7G-cap, which recruits eIF4G and eIF4A to form eIF4F. The 43S ribosome is recruited, scans the mRNA to find a start codon, and then recruits the 60S ribosomal subunit to form the 80S ribosome. (**b**) The internal ribosome entry sequence (IRES) within the 5′UTR experiences direct binding of 43S ribosome, followed by 60S ribosome joining. (**c**) TMG-mediated translation requires RNA helicase A/DHX9 (RHA) recognition of a three-way junction structure within the HIV 5′UTR for retention of the NCBP3/CBP80 heterodimer. Following 43S recruitment, either through ribosome scanning or internal ribosome entry, the 60S subunit joins to form the 80S complex, which initiates protein synthesis.

CBC exchange to the eIF4E m7G-cap-binding protein has been considered a rate-limiting step in translation control [37,38]. eIF4E activates the recruitment of eIF4G and eIF4A, and together these proteins comprise the tripartite eIF4F complex (Figure 2a). eIF4F joining to a 43S small ribosomal subunit activates the scanning of the 5′UTR for a suitable start codon; the 60S ribosomal subunit joins to form the 80S complex, and polypeptide synthesis begins.

### 3.2. Signal Transduction via mTOR Connects Metabolism with Global Translation

Early studies of translation control were focused on nutrient-rich conditions that enable cell growth and proliferation, e.g., the G1/S phase of the cell cycle [38]. But just as important to cell health and resilience is translation during nutrient-depleted conditions.

When resources are scarce, eIF4E binding proteins (4EBPs) bind eIF4E to block eIF4F assembly, repressing translation [39]. When amino acids, tRNA, and energy resources are high, the serine-threonine kinase mTOR phosphorylates 4EBPs to repress their activity, which allows protein translation to occur using the eIF4E pathway [40]. The term “global translation” refers to mTOR-driven protein synthesis, which accounts for ~65% of the total cellular translation [41,42,43,44,45].

mTOR control of eIF4E-mediated translation is critical for the host’s response to metabolic or environmental stress. Additionally, mTOR is intimately involved in cell cycle progression. During G1 and S, mTOR activity is high, 4EBP is repressed, and eIF4E translation occurs. G2 and M coincide with mTOR inhibition, 4EBP activation, and eIF4E translation abrogation. 4EBP activity thus serves as a checkpoint for the transition between these two metabolic states [38,39,40,46].

Logically, alternative pathways to initiate translation seem necessary: how else would cells adapt during environmental stress that inactivates eIF4E translation? How would cells maintain short-lived proteins for mitosis or resume eIF4E translation? In the case of Merkel cell polyomavirus, alternative means are used to hyper-phosphorylate 4EBP, which allows for eIF4E-mediated translation even when mTOR is inactive [47]. Some host mRNAs use alternative m7G-binding proteins to initiate translation. These include the DAP5/p97 isoform of eIF4G [48,49,50,51,52], FXR1a/PARN [53,54], eIF3d [55], and CTIF [56]. Some RNA viruses even antagonize eIF4F to suppress host translation yet maintain viral translation, e.g., picornaviruses. The predominant explanation for the continued translation is internal translation initiation on specific mRNAs.

### 3.3. Internal Ribosome Entry in Animal Viruses Overcomes Viral Antagonism of eIF4F

Internal ribosome entry is the targeted recruitment of the 43S ribosome to a specific region of an RNA known as the internal ribosome entry sequence (IRES) (Figure 2b). Several types of IRES exist. Typically, IRES motifs form complex structures for direct ribosome binding that are independent of mRNA caps. As presented in this Special Issue, elegant biochemical studies have classified the events for IRES translation in viruses [57]. For detailed information, the reader is referred to comprehensive reviews on IRES translation [18,58,59,60].

For HIV, evidence has been presented in favor of ribosome recruitment within the 5′UTR and gag coding region [18,61,62]. IRES activity in a bicistronic context was shown to be resistant to conditions that specifically inhibit eIF4F-dependent translation. These conditions include the presence of picornaviral 2A, HIV protease, or viral infections, all of which downregulate eIF4F activity [63,64]. IRES activity conveyed by the HIV 5′UTR was shown to be increased in cell cycle-arrested lysates [62,65,66]. Studies identified that HIV IRES activity exhibits cell-type specificity, with four-fold more reporter activity observed in Jurkat T cells compared to the HeLa fibroblast cell line [67].

### 3.4. HIV IRES Activity Is Measured in Bicistronic Reporter RNAs

Gendron et al. studied the HIV 5′UTR residues (+1–369) inclusive of the gag AUG and 30 nt of the gag sequence in transfected Jurkat T cells [68]. HIV 5′UTR residues +104 to 336 have IRES activity in bicistronic reporter constructs. Their investigation compared IRES activity between HIV and the hepatitis C virus (HCV). It was found that the HIV IRES produced only 20% as much translation as the HCV-positive control. HIV IRES activity increased 2-fold in response to the induction of oxidative stress, a condition that is relevant to the stress caused by HIV infection.

Deleting a portion of the IRES (nt +158–167) increased activity 2-fold; the authors speculated this was due to an internal ribosome entry negative element (IRENE) [68]. Notably, this same region was later shown to encompass a three-way junction structure necessary for HIV TMG-capping in immune cells [5]. The A140C structural mutation converts the three-way junction to a coaxial stem-loop structure that eliminates viral protein translation when eIF4E is inactive [69]. This outcome is the opposite of the IRENE phenotype identified in the bicistronic reporter mRNA by Gendron et al. [68], implicating that different molecular interactions operate in the context of the viral RNA and reporter RNA. We suggest studies are warranted to reconcile the different molecular interactions operating in the context of the TMG-capped viral RNA and the 5′UTR within a bicistronic reporter RNA.

## 4. RNA Helicase A (RHA) and NCBP3 as Novel Actors Within the TMG-Translation Pathway

### 4.1. RHA Is Necessary for Translation of Select mRNAs

Structural, biochemical, and genetic studies of the HIV 5′UTR revealed that the three-way junction structure (nt +135 to 235) is responsive to RNA helicase A (RHA/DHX9) [69]. Prior studies identified that RHA binds the HIV 5′UTR to facilitate translation and virion infectivity [69,70,71]. RHA downregulation or the A140C mutation redirects these transcripts to the mTOR-affected eIF4E-m7G-cap translation pathway, as shown in Singh et al. 2022 (Figures 4C and S6; Table S3 of reference [5]). Rev/RRE-dependent HIV RNAs retain RHA binding, whereas fully processed Rev/RRE-independent transcripts do not [5].

The RHA translation mechanism may be conserved across the *Retroviridae*. The 5′UTRs of HIV, human T cell leukemia virus type 1 (HTLV-1), spleen necrosis virus (SNV), feline leukemia virus, Mason-Pfizer monkey virus, and reticuloendotheliosis virus-A (RelA) are interchangeable for translation activity in HIV unspliced reporter RNA [71,72,73,74,75]. The 5′UTR sequences fail to support IRES activity in bicistronic RNAs, but HTLV-1, RelA, and SNV allow for translation during picornavirus-induced eIF4E downregulation [73]. SNV 5′UTR studies were the first to identify that RHA interaction with structural motifs of the retrovirus 5′UTRs bolsters translation [70,72,73,76].

Just as RHA binds the 5′UTR structure in retroviruses, it also associates with the 5′UTR of endogenous RNAs. Pull-down experiments identified that junD mRNA were among those that coprecipitated with RHA [70]. The JUND protein is an AP-1 transcription factor that is translated from unspliced mRNA. It has a complex 5′UTR but lacks IRES activity [77]. The identity of its non-canonical pathway was a longstanding gap in knowledge [78].

### 4.2. TMG-Cap Facilitates Non-Canonical Translation

In addition to junD, select selenoprotein mRNAs are translated independently of m7G-eIF4E activity, e.g., selT (thioredoxin reductase-like enzyme) and selR (methionine-R-sulfoxide reductase B1) [5,79]. The concept of TMG-cap on selenoprotein mRNAs arose from the work of Allmang and colleagues [17]. m7G-cap selenoprotein transcripts coprecipitate with eIF4E, whereas those with TMG-caps do not [17]. As a group, selenium-containing proteins are best known for their redox activity and anti-inflammatory properties, and their translation is stimulated by oxidative stress [80]. Selenoprotein mRNA is so named for its incorporation of selenocysteine (Sec), the 21st amino acid [81].

These transcripts harbor UGA termination codons that can be recoded to Sec codons. UGA recoding to the Sec codon requires the activity of the Sec insertion sequence (SECIS) in the 3′ UTR and the SECIS-binding protein (SBP2). The steps required for the Sec insertion begin with SBP2/SECIS interaction in the nucleus and CRM1-dependent nuclear export [82].

A later investigation of junD identified that it is also TMG-capped, which licenses its non-canonical translation. Furthermore, junD does not incorporate into RNPs that contain eIF4E; instead, it requires a TMG-cap for translation and associates with RNPs containing NCBP3/CBP80-RHA (Figure 2c) [83]. The TMG-capped selenoprotein mRNAs selT and selR are also associated with these distinct RNPs [5,79].

TGS1 was identified as a host protein that binds the Rev/RRE-dependent HIV RNA, and these transcripts obtain a TMG-cap [5,7,84]. These RNAs retained CBP80 in polysomes, whereas fully processed, Rev/RRE-independent HIV RNA was associated with eIF4E-containing polysomes [5]. RHA downregulation by siRNA eliminates TMG-cap from the viral transcripts, as well as junD. Co-immunoprecipitation assays targeting TMG-capped transcripts have shown RHA binding within the HIV and junD 5′UTR subverts exchange of CBC for eIF4E and is necessary for the formation of NCBP3/CBP80-TMG-mRNPs [70,71,77,83,85].

**Figure 3:**
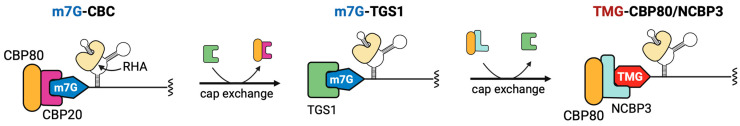
Model for hypermethylation of HIV m7G-cap to m2,2,7G TMG-cap. The m7G-cap-binding complex (CBC) is composed of heterodimeric CBP80 (orange) and CBP20, which directly binds m7G-cap (mauve). RNA helicase A/DHX9 (RHA, tan) recognizes a three-way junction structure within the HIV 5′ untranslated region and facilitates the exchange of CBC to TGS1 (green). The m7G-TGS1 complex is shown with RHA binding the three-way junction for m7G hypermethylation to m2,2,7G (red, TMG-cap). The TGS1 binding pocket does not accommodate the bulkier TMG structure and is released. The TMG-CBP80/NCBP3 complex results from the cap exchange to NCBP3 (blue) and CBP80.

The biological results support the following process to assemble TMG-mRNPs (Figure 3): (1) CBP20/80 binds m7G-capped mRNA to form CBC; (2) RHA tethers TGS1 near m7G-cap for its hypermethylation to TMG-cap; (3) TGS1’s cap-binding pocket cannot accommodate the new TMG-cap; and (4) NCBP3 replaces TGS1 to bind CBP80-associated TMG-cap.

### 4.3. Structural Studies Have Helped to Elucidate the Formation of These RNPs

TMG-cap has poor affinity toward m7G-cap-binding proteins eIF4E and CBP20/CBP80. Their binding of m7G-cap has been characterized as an aromatic sandwich motif that traps the guanosine in π-π stacking interactions [86]. Snurportin1, the snRNP-specific import adaptor protein, is the only known TMG-cap-binding protein [23]. Computational modeling of NCBP3 identified similarities to the cap-binding pocket of Snurportin [87]. Snurportin has been shown to bind the TMG-cap through π-π interactions between an aromate and the RNA’s transcription start site. CBP80/NCBP3 binding the TMG-cap is likewise proposed between the NCBP3 aromate and the RNA’s transcription start site through larger π-π stacking [87].

Structural studies help to understand the arrangement of NCBP3 in CBC RNPs. A 3.2 Å resolution cryoEM structure of the CBC in complex with the NCBP3 C-term domain (560–620) places the α-helical residues 574–594 of NCBP3 against CBP80 at its MIF4G domain [88]. One hydrogen bond between R105 of CBP20 and D574 of NCBP3 was observed in the CBP20/80-NCBP3 complex, although CBP80 alone can pull down NCBP3 [89]. Together, these data support that the CBP80/CBP20-NCBP3 tripartite complex represents a transition state when the m7G-cap is hypermethylated to TMG-cap and retains heterodimeric CBP80/NCBP3.

## 5. Adaptive Translation: Dual Initiation Strategies and eIF4E Repression

### 5.1. HIV-1 mRNAs Are Efficiently Translated When eIF4E Is Inactivated and Translation of Hosts Is Attenuated

In newly infected cells, proviral transcripts are fully processed, which removes the RRE motif. These RNAs, which encode the regulatory proteins Tat and Rev, retain their m7G-cap and are translated by the eIF4E pathway [90]. Tat binds the Transactivation Response (TAR) element to trans-activate proviral transcription. On the other hand, Rev binds the HIV RRE motif and leads to an increase in HIV RNA containing this element. Rev/RRE trans-activates the nuclear export of these RNAs in a CRM1-dependent manner [91]. The Rev/RRE-dependent transcripts template the synthesis of viral accessory proteins that modify virus–host interplay (Vpr, Vif, Vpu) and structural proteins that are components of progeny virions (Gag, Gag-Pol, Env).

Viral protein R (Vpr) activates 4EBP to repress eIF4E activity, which inhibits host protein translation by 65%, as well as the translation of Rev/RRE-independent transcripts, yet HIV virion protein synthesis is maintained [92]. Rev/RRE-dependent transcripts display an unexpected property; rather than obtaining eIF4E, these RNAs retain CBP80 in polysomes and thus use a different translation pathway [92]. This led to the hypothesis that the CBP80-containing polysomes coincide with viral protein synthesis during eIF4E inactivation. By altering the presence of RRE, HIV can utilize dual translation initiation strategies.

HIV translation thus adapts to the host cell environment to balance viral protein expression with cell survival. How HIV contributes to the metabolic reprogramming of hosts has been an active area of research [93,94,95,96,97,98,99,100,101,102,103,104,105]. Increased metabolic activity engenders T cells and monocytes that are more susceptible to HIV-1 infection [100,106].

### 5.2. Translation Machinery of Hosts Is Fundamentally Responsive to Metabolism Shifts

New findings demonstrate that HIV co-opts the interplay of m7G- and TMG-translation pathways to moderate viral protein production in favor of host cell survival. HIV requires host resources and translation mechanisms to synthesize its proteins. Early replication events, including reverse transcription, integration, and provirus transcription [96,107,108,109,110,111,112,113,114], require mTOR activity present in activated T cells.

The transition of Tcells from Effector to Memory to Reactivated states coincides with mTOR inhibition and restoration, respectively, of the eIF4E-m7G translation pathway [115]. Experiments have documented that Tat, Rev, and Nef protein synthesis in CD4+ T cells is curtailed by supplementation with an mTOR inhibitor and restored by the replacement of fresh medium [5,83]. These results indicate that virion protein synthesis can continue as T cells transition to a memory state, even when viral regulatory proteins turnover and transactivation of new proviral transcription is curtailed.

## 6. Conclusions

To produce its complicated proteome, HIV utilizes both mTOR-dependent and -independent strategies for translation initiation. While IRES is generally considered the alternative to the mTOR pathway of protein synthesis, new evidence indicates that the novel TMG-cap translation pathway is a new explanation for translation that is sustained when eIF4F is inactive. 

This article has presented evidence that the TMG-cap pathway is a critical component of biphasic HIV protein synthesis. Since HIV fully processed mRNAs are m7G-capped and licensed for translation by eIF4F mRNPs, regulatory proteins cease translation when eIF4E is inactive. TMG-capping of the Rev/RRE-dependent RNAs licenses assembly of a novel translation mRNP composed of NCBP3/CBP80 and RHA. This specialized translation pathway ensures virion protein translation when eIF4E is inactive. We postulate that the interplay between these translation pathways strategically moderates HIV regulatory protein production to tune infectious virion production to favor host immune cell survival. 

The characterization of other components of the NCBP3/CBP80-RHA mRNP that is ongoing will provide clues to a comprehensive understanding of the translation of host TMG-capped mRNAs. Soto-Rifo and colleagues identified that CBP80 interaction with Rev promotes nuclear export and translation of the HIV unspliced RNA [116]. Rev also associates with CTIF, the CBP80/20-dependent translation initiation factor associated with nonsense-mediated decay [117]. The CTIF overexpression inhibits Gag synthesis in a dominant negative manner [116]. We speculate that NCBP3 may take the place of CTIF to bridge Rev-CBP80 and the 43S ribosomal subunit for steady state translation unaffected by mTOR [118]. With the development of ribosome profiling of HIV mRNAs [119,120,121], the relationship between the dual translation mechanisms licensed by TMG-cap or m7G-cap can now be investigated with great precision. 

**Figure 4:**
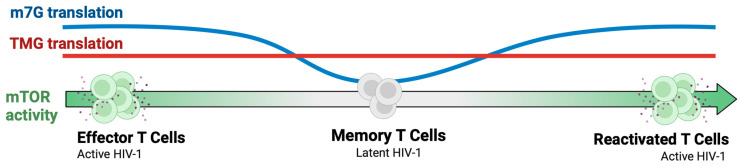
Model for the HIV translation shift across T cell states. mTOR activity supports provirus formation, transcription and processing of viral transcripts. Translation of m7G-capped mRNA (blue) is synchronous with mTOR activity, whereas TMG-capped viral and host mRNAs engage in constitutive translation (red). As metabolic resources decline, mTOR activity wanes (green arrow transition to gray) and Effector T cells progress toward a memory state. Suppressed translation of HIV m7G-capped mRNAs to regulatory proteins diminishes new viral gene expression, diminishing capacity for virion production. Constitutive translation of residual TMG-capped Rev/RRE-dependent viral mRNAs prolongs the generation of virions despite mTOR inhibition, seeding the latent viral reservoir. Restored mTOR activity in reactivated Effector T cells reactivates productive viral infection and dissemination. Gray dots, progeny virions.

Some people living with HIV exhibit significantly reduced plasma concentrations of essential amino acids relative to healthy controls (*p* < 0.05), indicative of metabolic stress [122]. Low resource abundance leads to mTOR inhibition, and hosts abrogate eIF4F-dependent translation while specialized translation continues (Figure 4). A new perspective on viral pathogenesis is emerging that HIV-induced metabolism shifts and gene expression remodels can moderate viral production in tune with the immune cell population susceptible to infection [100].

## Data Availability

New data were not part of this manuscript.
